# Integrative analysis of morphological traits and targeted flavonoid profiles reveals chemotaxonomic signatures in *Abelmoschus* species

**DOI:** 10.3389/fpls.2026.1860412

**Published:** 2026-08-03

**Authors:** Yoon-Jung Lee, Bum-Soo Hahn, Seong-Hoon Kim, Aejin Hwang, Hyeonseok Oh, Young-Wang Na

**Affiliations:** National Agrobiodiversity Center, National Institute of Agricultural Sciences, Rural Development Administration (RDA), Jeonju, Republic of Korea

**Keywords:** *Abelmoschus*, chemodiversity, chemotaxonomy, crop wild relatives, okra, plant genetic resources, quercetin glycosides, UPLC–MS/MS

## Abstract

The genus *Abelmoschus* includes cultivated okra (*A. esculentus*) and diverse wild species and is of considerable value as an edible and medicinal resource. However, accurate species identification remains difficult due to their morphological similarity and complex taxonomic history. In this study, we compared 14 agronomic traits and the composition of eight target flavonoids in pods across 117 accessions representing seven *Abelmoschus* species grown under the same field conditions and evaluated their potential utility as chemotaxonomic indicators. Morphological trait analysis mainly separated the cultivated species from the wild species, but provided limited resolution among closely related or morphologically similar taxa. However, flavonoid profiling using UPLC–MS/MS revealed compositional differences that were broadly consistent with species identities in the accessions examined. In particular, hyperoside and rutin exhibited the strongest discriminatory signals among the compounds examined. Multivariate analysis further indicated that flavonoid profiles tended to separate the morphologically similar *A. esculentus* and *A. caillei* and supported the presence of three major compositional clusters across the analyzed accessions. Although the number of accessions was limited for some species, these results suggest that targeted flavonoid profiling can complement conventional morphology-based classification. These findings provide a basis for the chemotaxonomic evaluation of *Abelmoschus* genetic resources and may inform future breeding strategies and functional resource utilization.

## 1 Introduction

The genus *Abelmoschus* comprises tropical and subtropical plants in the family Malvaceae, many of which have been widely used as an edible, medicinal, and industrial resource. This genus includes several cultivated and wild species, and its flowers, leaves, seeds, and pods are used for various purposes. Okra (*Abelmoschus esculentus*), the best-known cultivated species, is a major vegetable crop grown worldwide. *A. caillei*, commonly referred to as West African okra, is an important crop in the regional food culture of Western and Central Africa (Osawaru and Dania-Ogbe, 2010). *Abelmoschus manihot* has long been used medicinally in traditional East Asian medicine, particularly its flowers (Guo et al., 2015; Li et al., 2021). *Abelmoschus moschatus* has been used as a source material in the cosmetic industry because of its seeds, whereas *Abelmoschus ficulneus* has been used as a medicinal herb in traditional Indian medicine (Pawar and Vyawahare, 2017; Dashputre and Bandawane, 2021).

Despite this economic and pharmacological importance, the genus *Abelmoschus* has unclear species boundaries and complex nomenclatural treatments (Patil et al., 2015a). This taxonomic instability is well illustrated by the treatment of *Abelmoschus caillei* and *Abelmoschus tetraphyllus*. *A. caillei* was initially described as *Hibiscus manihot* var. *caillei* (Chevalier, 1940) and was later transferred to *Abelmoschus* as *Abelmoschus caillei* (Stevels, 1988). In contrast, *A. tetraphyllus* has been treated in some taxonomic systems as a variety or subspecies of *A. manihot* (Hochreutiner, 1924; Van Borssum Waalkes, 1966), whereas other studies have recognized it at the species level (Siemonsma, 1982; Hamon and Van Sloten, 1995; Werner et al., 2016). Species delimitation in *Abelmoschus* is further complicated by its complex nomenclatural history and the absence of clear morphological diagnostic characters (Patil et al., 2015a). In addition, *A. esculentus* and *A. caillei* share several morphological traits, which may cause taxonomic problems during field collection and morphology-based identification (Essilfie et al., 2010). Therefore, morphological traits alone may not provide stable criteria for species discrimination in *Abelmoschus* germplasm. Additional quantitative and reproducible taxonomic indicators are thus needed to complement morphology-based classification.

To address these limitations, molecular phylogenetic and genomic studies have been conducted; however, species boundaries in *Abelmoschus* remain incompletely resolved. Previous phylogenetic analyses have shown limited resolution in separating closely related taxa such as *A. caillei* and *A. esculentus*, and have also revealed cases in which molecular relationships did not correspond to morphological affinities (Werner et al., 2016). Recent cytogenetic and whole-genome studies further suggest that hybridization and allopolyploidy may be important contributors to the biological complexity of *Abelmoschus*, particularly in okra and its related taxa, and may have influenced its speciation and genome architecture (Wang et al., 2023a; Nieuwenhuis et al., 2024). Consequently, morphological traits and available molecular evidence are not always sufficient to reliably differentiate closely related *Abelmoschus* species, underscoring the need for complementary approaches such as metabolite-based chemical profiling.

Such profiling has been applied to distinguish closely related plant species, accessions, and varieties (Farag et al., 2016; Afzan et al., 2019). Flavonoids are particularly informative in this regard: their profiles often differ among species or taxa in compound occurrence, relative abundance, and glycosylation patterns (Güzel et al., 2011; Chakarova et al., 2025). In *Daphne* and *Wikstroemia*, for instance, flavonoid composition retained genus- and species-level signals even after ecological variation was taken into account, reinforcing its value alongside morphological and molecular data (Son et al., 2025). Comparable MS-based phytochemical studies in *Sorbus*, *Ipomoea*, and *Momordica* have associated flavonoid-containing profiles with hybrid origin, species boundaries, and species-dependent chemical variation (Korić et al., 2025; Salim et al., 2026; Ramabulana et al., 2021). Earlier work pointed in the same direction, using flavonol glycosides and sugar-moiety patterns for species and section classification in *Rosa* (Sarangowa et al., 2014) and identifying isorhamnetin- and tricin-based glycosides as discriminant metabolites among *Amaranthus* species (Abdel-Moez et al., 2023). Together, these cases show that flavonoid profiles can yield quantifiable chemical traits that complement morphological and molecular characters in species and germplasm discrimination.

Despite these examples in other genera, chemical studies of the genus *Abelmoschus* have mainly focused on the identification of bioactive constituents and the evaluation of pharmacological activities in a limited number of species, particularly *A. esculentus* and *A. manihot*. Comparative chemotaxonomic evaluations across multiple species remain limited. In *A. esculentus*, catechin, epicatechin, rutin, quercetin, procyanidin B1/B2, and quercetin-derived flavonol glycosides have been reported as major flavonoid and related phenolic constituents (Arapitsas, 2008; Yang et al., 2022). In *A. manihot*, flavonoids such as rutin, hyperoside, and hibifolin have also been identified (Luan et al., 2020; Diao et al., 2023). By contrast, detailed identification and quantification of individual flavonoids remain limited in wild or underutilized species such as *A. caillei*, *A. ficulneus*, *A. tetraphyllus*, and *Abelmoschus tuberculatus* (Pascal et al., 2018; Mohite and Gurav, 2019; Kaur et al., 2024). This species-level imbalance limits a comprehensive understanding of the chemical diversity within *Abelmoschus* and hinders the application of chemotaxonomic approaches to germplasm classification and utilization.

In the present study, we combined morphological characterization with UPLC–MS/MS-based flavonoid quantification for seven *Abelmoschus* species (*A. esculentus*, *A. caillei*, *A. manihot*, *A. moschatus*, *A. tetraphyllus*, *A. tuberculatus*, and *A. ficulneus*) grown under the same field conditions. Flavonoids, a major phenolic class repeatedly reported in *Abelmoschus* species, were selected as chemotaxonomic markers, and quantitative profiles of eight reliably detected flavonoids were used as complementary biochemical indicators. Overall, we aimed to characterize differences in flavonoid composition among *Abelmoschus* species and propose chemotaxonomic indicators that may support species identification and the systematic utilization of genetic resources within this genus.

## 2 Materials and methods

### 2.1 Plant materials

*Abelmoschus* accessions maintained at the National Agrobiodiversity Center, Jeonju, Republic of Korea, were used in this study. Detailed accession information is provided in **Supplementary Table 1**. The accessions were cultivated under open-field conditions in the experimental field of the National Agrobiodiversity Center. Seeds were sown directly in the field on May 12, 2024, and 10 plants were grown per accession. All seven species were grown in the same field under identical agronomic management conditions. Pods were harvested at the immature edible stage immediately before fibrous hardening (before fiber lignification). In *A. esculentus*, this stage corresponds to approximately 6–7 days after anthesis. Notably, the harvest timing for the wild species was adjusted according to the growth rate to obtain pods at a comparable developmental stage. In total, 117 accessions, including 98 of *A. esculentus*, two of *A. caillei*, four of *A. tetraphyllus*, five of *A. manihot*, three of *A. moschatus*, four of *A. tuberculatus* and one of *A. ficulneus*, were analyzed.

### 2.2 Comparison of key agronomic traits among species

In this study, we evaluated the following 14 morphological and agronomic traits for interspecific comparison: cotyledon length and width, hypocotyl length, plant height, stem diameter, leaf blade length and width, petiole length, number of petals, petal length, fruit length, fruit width, fruit weight, and number of locules. Among the 10 plants established per accession, three vigorous and phenotypically uniform individuals were selected for trait measurements. Vegetative traits were measured at a common developmental stage, corresponding to peak vegetative growth. Fruit traits were measured on pods harvested after the cessation of elongation growth but before hardening. Stem diameter was measured using digital calipers, and linear traits were measured using a ruler.

### 2.3 Taxonomic verification and re-identification of misclassified accessions

**Figure 1:**
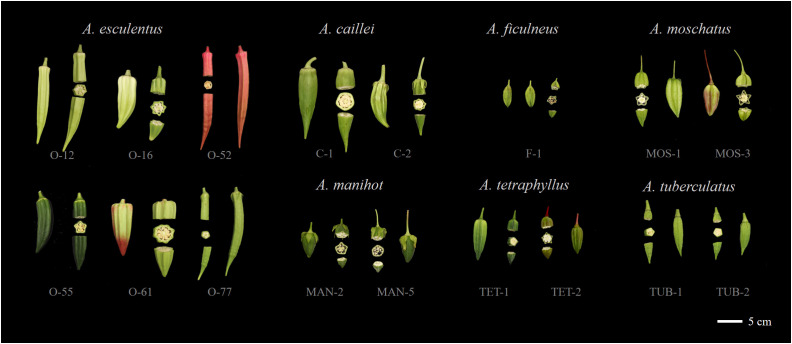
Morphological diversity of fruit pods among seven *Abelmoschus* species. Images of immature pods are shown for visual comparison of pod shape, surface features, and color variation among species. Species names, analysis IDs, and a 5-cm scale bar are indicated in the figure.

**Figure 2:**
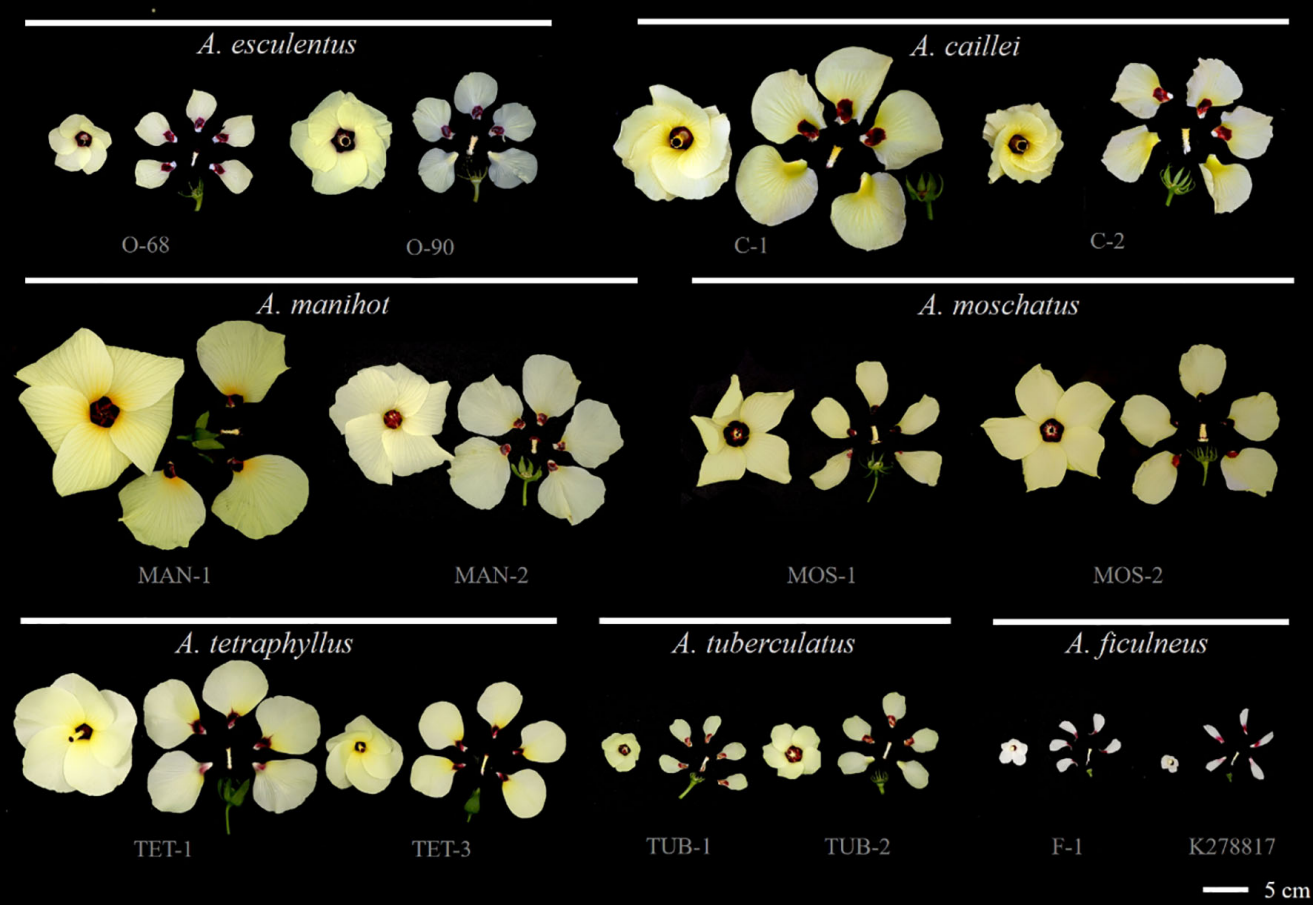
Floral morphology of seven *Abelmoschus* species. Two representative accessions are shown for each species. Each accession included an intact flower and dissected floral organs (petals, reproductive column, and calyx). Accession K278817 (*A. ficulneus*) is for visual comparison and was not used in any quantitative analyses. Scale bar, 5 cm.

Taxonomic verification of all 117 accessions was conducted by comparing passport-data species names with observed morphological traits and published diagnostic descriptions for *Abelmoschus* species. Species identification was based primarily on floral characters, epicalyx morphology, fruit shape and dehiscence pattern, and seed and vegetative traits (Yadav et al., 2014), with additional support from numerical taxonomic evidence for the genus (Patil et al., 2015b). Four previously misclassified accessions were re-identified and classified accordingly (**Supplementary Table 1**). Representative images of immature pods and floral morphology were used to facilitate interspecific comparisons (**Figures 1**, **2**).

### 2.4 Preparation of sample solution for ultra-performance liquid chromatography–tandem mass spectrometry

For flavonoid analysis, pods from the three most phenotypically uniform individuals within each accession were pooled to generate one accession-level composite sample. Pooled fresh pods were freeze-dried, pulverized, and extracted with 80% ethanol at a sample-to-solvent ratio of 1:20 (w/v) at 40 °C for 1 h; the extraction conditions were adapted from Wang et al. (2023b), with minor modifications. After centrifugation at 4,000 × g for 15 min, the supernatant was filtered through a 0.2 μm syringe filter to obtain the crude extract. For UPLC–MS/MS analysis, 200 μL of the crude extract was mixed with 800 μL of 80% ethanol containing galangin (0.5 ppm) as an internal standard, and the final solution was filtered through a 0.2 μm membrane filter before injection.

### 2.5 UPLC–MS/MS instrumentation and chromatographic conditions

Chromatographic separation was performed using an ACQUITY UPLC I-Class PLUS system (Waters, Milford, MA, USA) coupled to a Xevo TQ-S micro triple-quadrupole mass spectrometer (Waters). Analytes were separated on an ACQUITY UPLC CSH C18 column (150 × 2.1 mm, 1.7 μm; Waters) at 40 °C with a flow rate of 0.3 mL/min and an injection volume of 1 μL. The mobile phases were 0.1% formic acid in water (A) and 0.1% formic acid in methanol (B). The gradient program was as follows: 0.0 min, 90% A/10% B; 0.2 min, 70% A/30% B; 8.0 min, 30% A/70% B; 10.0 min, 10% A/90% B; 15.0 min, 10% A/90% B; 20.0 min, 90% A/10% B for re-equilibration.

### 2.6 Mass spectrometric conditions and MRM transitions

The ESI source was operated in the positive ion mode with the following settings: capillary voltage, 2.5 kV; sampling cone voltage, 30 V; source temperature, 150 °C; desolvation temperature, 600 °C; and desolvation gas flow (nitrogen), 1000 L/h. Data were acquired in the non-scheduled multiple reaction monitoring (MRM) mode. Quantification was performed using a single MRM transition per analyte. Target flavonoids were identified by matching their retention times to those of authentic standards and quantified using the corresponding optimized MRM transition (**Supplementary Table 3**).

### 2.7 Quantification and data processing

Quantification was based on calibration curves constructed from authentic standards, with galangin as the internal standard. Calibration curves were constructed by plotting the analyte-to-internal standard peak area ratios against the nominal concentrations. Raw data were processed using MassLynx and TargetLynx (TargetLynx XS Application Manager, Waters). Each extract was analyzed using three technical injections, and the concentrations were reported as ppm (mg/L) in the final analytical solution prior to injection. Calibration characteristics, including LOD and LOQ, are provided in **Supplementary Table 4**.

### 2.8 Statistical analysis

Agronomic traits were evaluated using three biological replicates per accession, and replicate means were used for analysis. For flavonoid analysis, each accession was represented by one composite extract, and the mean of three technical UPLC–MS/MS injections was used. Species differences in flavonoid concentrations were assessed using Kruskal–Wallis tests followed by Wilcoxon rank-sum *post hoc* tests, with Benjamini–Hochberg correction applied across compounds and within each compound, respectively. Differential accumulation was defined as BH-adjusted q < 0.05 and |log_2_FC| ≥ 1.0, with ND values treated as zero.

PCA was performed separately on z-score-standardized agronomic trait and flavonoid composition data. Presence/absence-based PCoA was conducted using Jaccard dissimilarity, and species-associated structure was evaluated by PERMANOVA with 999 permutations. Congruence between the z-score-standardized agronomic trait and flavonoid composition datasets was assessed using Procrustes analysis of their PCA ordinations and, separately, a Pearson Mantel test on Euclidean distance matrices, both with 999 permutations. Median-based PERMDISP and a sensitivity analysis excluding species represented by fewer than three accessions were performed to evaluate the robustness of the PERMANOVA result. Heatmap visualization and k-means clustering were applied to the flavonoid data, with the optimal k determined by silhouette analysis. All analyses were performed in R version 4.5.2 using ggplot2, factoextra, ggcorrplot, vegan, Hmisc, and pheatmap.

## 3 Results

### 3.1 Morphological differentiation of cultivated and wild *Abelmoschus* species

**Figure 3:**
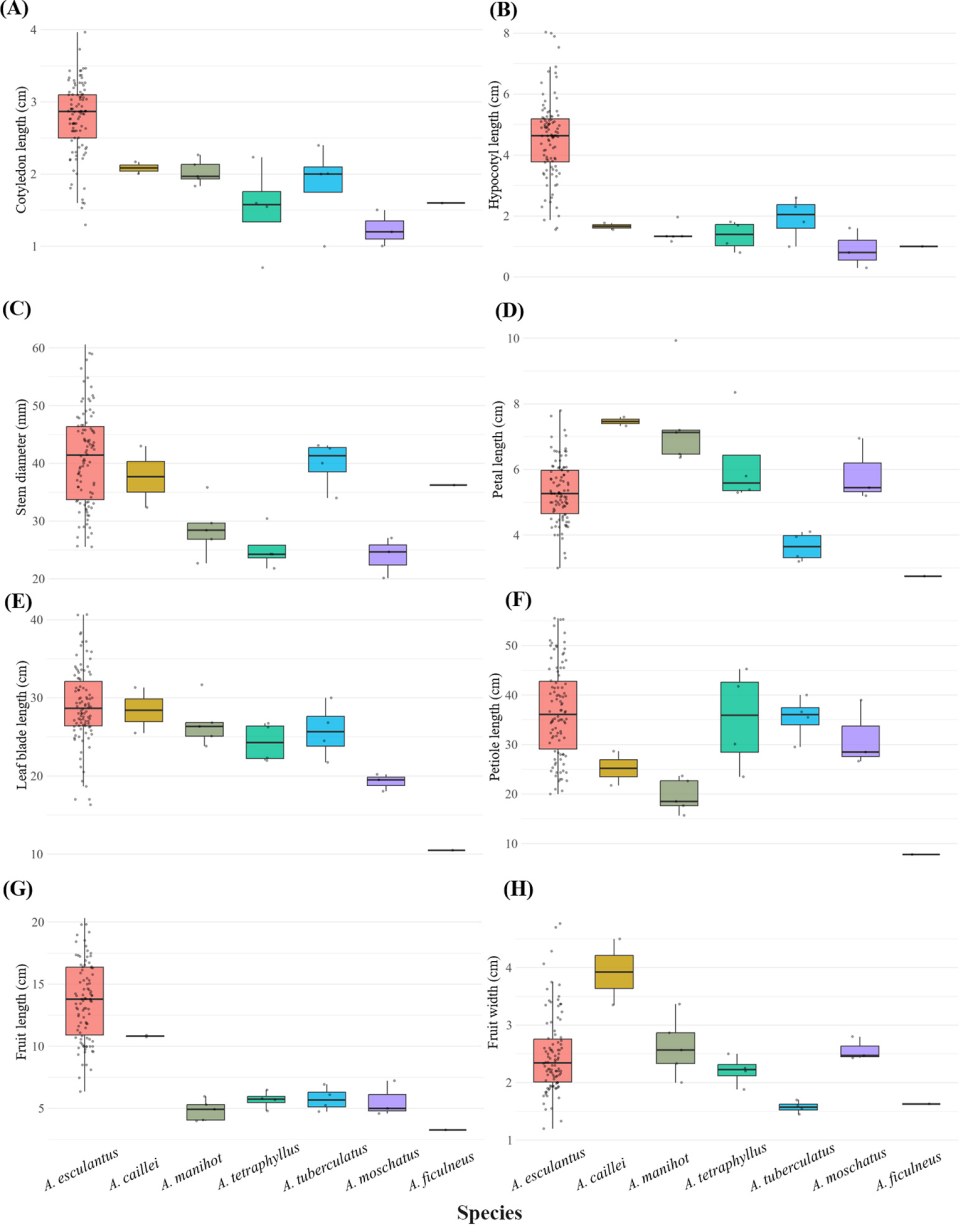
Comparison of morphological traits among seven *Abelmoschus* species. Box plots show the distributions of eight morphological traits: **(A)** cotyledon length (cm), **(B)** hypocotyl length (cm), **(C)** stem diameter (mm), **(D)** petal length (cm), **(E)** leaf blade length (cm), **(F)** petiole length (cm), **(G)** fruit length (cm), and **(H)** fruit width (cm).

In this study, we compared 14 morphological and agronomic traits across 117 accessions representing seven species of *Abelmoschus*. Summary statistics for each species are provided in **Supplementary Table 5**. In addition, the distributions of eight representative traits selected based on their contribution to among-species variation were visualized as boxplots (**Figures 3A–H**).

Among the measured traits, fruit size-related characteristics showed the clearest differences between the cultivated species, *A. esculentus* and *A. caillei*, and the wild species. In particular, mean fruit length was markedly shorter in the wild species (3.27–5.76 cm) than in the cultivated species (*A. esculentus*, 13.86 ± 3.41 cm; *A. caillei*, 10.83 ± 0.11 cm; **Figure 3G**). Similarly, fruit weight was higher in the cultivated species (31.74 ± 13.84 g in *A. esculentus* and 40.23 ± 12.83 g in *A. caillei*) than in the wild species (2.17–6.85 g). *A. caillei* had the highest mean fruit width and locule number (3.93 ± 0.81 cm and 8.25 ± 1.06, respectively), followed by *A. esculentus* (2.51 ± 0.74 cm and 6.89 ± 1.17, respectively). Among the wild species, *A. tuberculatus* had the narrowest fruit width (1.58 ± 0.10 cm), whereas *A. ficulneus* showed the lowest values for fruit length (3.27 ± 0.27 cm) and fruit weight (2.17 ± 0.25 g), indicating the smallest fruit size among the species examined (**Supplementary Table 5**). Differences among species were also observed in seedling traits. Hypocotyl length was markedly longer in *A. esculentus* (4.56 ± 1.32 cm) than in the other species (0.90–1.93 cm; **Figure 3B**). In addition, *A. esculentus* and *A. moschatus* exhibited the longest and shortest cotyledon length, respectively (2.77 ± 0.51 vs. 1.23 ± 0.25 cm; **Figure 3A**). Although *A. caillei* resembled *A. esculentus* in terms of enlarged fruit traits, its seedling size traits were more similar to those observed in the wild species.

In addition, species-specific variations were observed in floral and vegetative growth traits. Petal length was relatively long in *A. caillei* (7.47 ± 0.19 cm) and *A. manihot* (7.42 ± 1.45 cm), but short in *A. tuberculatus* (3.65 ± 0.38 cm) and *A. ficulneus* (2.75 ± 0.10 cm) (**Figure 3D**). Although the number of petals was five in most species, *A. esculentus* exhibited variations in petal number from five to eight, with a mean of 5.28 ± 0.52. **Figure 2** shows species-specific differences in qualitative floral traits, including flower color.

Regarding vegetative traits, *A. tuberculatus* showed the greatest plant height (239.92 ± 45.47 cm) and one of the largest stem diameters (39.94 ± 3.62 mm) among the species examined, despite its relatively small fruit size. Petiole length was relatively long in *A. esculentus* (36.72 ± 9.22 cm), *A. tetraphyllus* (35.15 ± 10.11 cm), and *A. tuberculatus* (35.40 ± 4.37 cm). In contrast, *A. ficulneus* exhibited the shortest petiole length (7.80 ± 0.80 cm) and leaf blade length (10.50 ± 0.70 cm), consistent with its generally reduced morphology across vegetative organs and fruit traits (**Figures 3E, F**). Together, these results indicate that morphological differentiation among the sampled *Abelmoschus* species was driven primarily by fruit-size-related traits, whereas seedling, floral, and vegetative traits revealed additional species-specific deviations from this main pattern.

### 3.2 Principal component structure of morphological variation

In the PCA score plot, *A. esculentus* accessions were broadly dispersed but mainly distributed in the positive region of PC1 and remained largely distinct from most wild species, whereas the two *A. caillei* accessions were located near the PC1 origin but differed from each other along the PC1 axis, placing them in an intermediate position between *A. esculentus* and the wild species.

Based on the loading patterns of individual trait vectors, the positive direction of PC1 was strongly associated with petiole length, leaf blade length and width, fruit weight and width, number of locules, and stem diameter, indicating that PC1 primarily reflected variations in the size of fruits and vegetative organs. In contrast, petal length was positioned in an intermediate region without a clear bias toward either PC1 or PC2, suggesting a relatively limited contribution to separation along the first two principal component axes compared with the other major size-related traits.

For PC2, petiole length, plant height, leaf blade size-related traits, and fruit width were oriented toward the positive direction, whereas cotyledon length, cotyledon width, hypocotyl length, and fruit length were oriented toward the negative direction. Most wild species were concentrated in the PC1-negative and PC2-positive region, although their positions along PC2 varied among species. The single *A. ficulneus* accession deviated from this pattern, being positioned toward the negative side of PC2 and apart from the other wild species. Given the limited sampling of *A. caillei* and *A. ficulneus*, these positions represent relative tendencies among the sampled accessions. Overall, the morphological PCA resolved a broad cultivated–wild contrast mainly along PC1 but did not provide complete species-level separation.

### 3.3 Species-associated flavonoid composition patterns across *Abelmoschus* species

Targeted UPLC–MS/MS analysis of pod extracts from seven *Abelmoschus* species was performed using 12 flavonoid standards. Four compounds were not detected in any accession, namely quercetagetin 7*-O-*glucoside, quercetin 3*-O-*(6′′*-O-*acetyl)-glucoside, hibifolin, and isorhamnetin. Quantification was therefore performed for the eight detected compounds, including catechin, epicatechin, quercetin 3*-O-*sambubioside, quercetin 3*-O-*gentiobioside, rutin, hyperoside, isoquercitrin, and quercetin 3*-O-*(6′′*-O-*malonyl)-glucoside (**Table 1**; **Supplementary Table 3**). The eight quantified compounds comprised two flavan-3-ols and six quercetin-derived flavonol glycosides, including a malonylated quercetin glucoside (**Supplementary Table 2**).

**Table 1:** Species-specific concentrations of targeted flavonoids in pods of seven *Abelmoschus* species determined by UPLC–MS/MS.

Species (n)	Catechin	Epicatechin	Quercetin 3*-O-*(Sambu)	Quercetin 3*-O-*(Gentio)	Rutin	Hyperoside	Isoquercitrin	Quercetin 3*-O-*(Malonyl-Glc)
A. esculentus (n = 98)	0.51 ± 1.06 (0.00–4.05)	21.69 ± 9.68 (0.00–57.75)	3.62 ± 1.76 (0.65–10.13)	21.00 ± 7.25 (6.47–45.85)	0.07 ± 0.34 (0.00–2.46)	0.03 ± 0.24 (0.00–2.02)	10.49 ± 3.20 (5.11–22.06)	5.37 ± 2.49 (0.83–15.73)
A. caillei (n = 2)	ND	14.89 ± 5.07 (11.30–18.47)	22.63 ± 4.47 (19.47–25.79)	ND	1.53 ± 2.17 (0.00–3.06)	0.11 ± 0.15 (0.00–0.22)	10.47 ± 2.12 (8.97–11.97)	2.53 ± 0.07 (2.48–2.57)
A. manihot (n = 5)	ND	ND	1.56 ± 2.24 (0.00–4.83)	0.45 ± 0.51 (0.00–1.01)	2.76 ± 1.33 (1.01–4.19)	0.96 ± 0.57 (0.22–1.48)	2.71 ± 1.31 (0.97–4.23)	1.33 ± 0.62 (0.55–1.98)
A. tetraphyllus (n = 4)	0.35 ± 0.71 (0.00–1.41)	21.00 ± 5.70 (12.59–24.83)	10.79 ± 4.48 (5.49–14.94)	27.43 ± 4.56 (21.28–31.96)	6.32 ± 0.58 (5.65–7.05)	0.62 ± 0.10 (0.53–0.72)	34.14 ± 4.80 (28.29–38.50)	3.84 ± 0.81 (3.25–5.00)
A. tuberculatus (n = 4)	ND	9.78 ± 6.59 (0.00–14.44)	ND	ND	6.96 ± 3.66 (3.35–12.05)	0.68 ± 0.63 (0.05–1.55)	3.02 ± 2.23 (0.57–5.81)	5.36 ± 2.46 (2.00–7.78)
A. moschatus (n = 3)	ND	35.38 ± 16.26 (22.55–53.67)	10.04 ± 3.20 (7.85–13.72)	ND	7.21 ± 4.43 (3.19–11.95)	0.71 ± 0.58 (0.13–1.29)	11.85 ± 3.91 (9.13–16.34)	0.04 ± 0.06 (0.00–0.11)
A. ficulneus (n = 1)	ND	41.56 †	34.88 †	54.85 †	0.16 †	ND	33.09 †	11.04 †

quercetin 3*-O-*(sambu), quercetin 3*-O-*sambubioside; quercetin 3*-O-*(gentio), quercetin 3*-O-*gentiobioside; quercetin 3*-O-*(malonyl-glc), quercetin 3*-O-*(6″*-O-*malonyl)-glucoside. ND, not detected. † Single value; standard deviation and range are not applicable as *A. ficulneus* is represented by only one accession (n = 1). ND values were treated as zero for statistical analyses.

Data are presented as mean ± standard deviation, with the minimum and maximum values in parentheses. Concentrations are expressed as ppm in the final analytical solution prior to injection.

Occurrence patterns showed qualitative differences among species. Rutin, isoquercitrin, and quercetin 3*-O-*(6′′*-O-*malonyl)-glucoside were detected in all species, whereas the remaining compounds showed more restricted distributions. Catechin occurred only in *A. esculentus* and *A. tetraphyllus*. Epicatechin was detected in all species except *A. manihot*. Quercetin 3*-O-*sambubioside was detected in all species except *A. tuberculatus*. Hyperoside was detected in all species except *A. ficulneus*. Quercetin 3*-O-*gentiobioside showed a narrower distribution than quercetin 3*-O-*sambubioside and was detected only in *A. esculentus*, *A. manihot*, *A. tetraphyllus*, and *A. ficulneus*. Within the targeted panel, *A. tuberculatus* was thus characterized by the absence of both quercetin 3*-O-*sambubioside and quercetin 3*-O-*gentiobioside.

Quantitatively, the two cultivated species examined showed contrasting quercetin glycoside profiles. In *A. esculentus* (n = 98), the most abundant compounds were epicatechin (21.69 ± 9.68 ppm), quercetin 3*-O-*gentiobioside (21.00 ± 7.25 ppm), and isoquercitrin (10.49 ± 3.20 ppm). The mean concentration of quercetin 3*-O-*gentiobioside was approximately six times that of quercetin 3*-O-*sambubioside (3.62 ± 1.76 ppm), indicating a gentiobioside-dominant pattern. By contrast, quercetin 3*-O-*gentiobioside was not detected in *A. caillei* (n = 2), in which quercetin 3*-O-*sambubioside reached a comparatively high level (22.63 ± 4.47 ppm), suggesting a sambubioside-dominant pattern.

Kruskal–Wallis tests indicated significant species-associated variation for seven of the eight quantified flavonoids, with catechin being the only exception (q = 0.662; **Table 2**). Hyperoside (ϵ^2^ = 0.808) and rutin (ϵ^2^ = 0.691) had the largest effect sizes, although their absolute concentrations were low in several species. In pairwise Wilcoxon tests, significant differences were concentrated in comparisons involving *A. esculentus* (**Supplementary Table 6**): hyperoside and rutin accounted for the largest number of significant contrasts, whereas the other quercetin glycosides and epicatechin showed more comparison-specific differences.

**Table 2:** Species-associated variation in flavonoid compounds across seven *Abelmoschus* species.

Compound	Kruskal–Wallis H	Kruskal–Wallis q	Effect size (ϵ^2^)	Significant pairs (n)
Hyperoside	94.89	2.336e-17	0.808	5
Rutin	82.02	5.473e-15	0.691	5
Quercetin 3-O-(Gentio)	41.86	5.214e-07	0.326	3
Quercetin 3-O-(Sambu)	37.57	2.731e-06	0.287	3
Isoquercitrin	36.44	3.621e-06	0.277	3
Quercetin 3-O-(Malonyl-Glc)	29.25	7.270e-05	0.211	2
Epicatechin	25.94	2.614e-04	0.181	1
Catechin	4.11	6.620e-01	0.000	0

Kruskal–Wallis q-values were adjusted across the eight quantified flavonoid compounds using the Benjamini–Hochberg method. Effect size was estimated using epsilon-squared (ϵ^2^). “Significant pairs” indicates the number of species-pair comparisons satisfying both within-compound Wilcoxon BH q < 0.05 and |log_2_FC| ≥ 1.0, as detailed in **Supplementary Table 6**.

Given the marked imbalance in accession numbers, the quantitative pairwise results should be interpreted with caution. To complement abundance-based comparisons, we further evaluated the occurrence patterns described above using binary presence/absence profiles. PCoA based on Jaccard dissimilarity supported a species-associated qualitative structure (**Supplementary Figure 1**), and exploratory PERMANOVA showed significant differences among species (R^2^ = 0.740, F = 52.23, p = 0.001). Median-based PERMDISP did not indicate significant dispersion heterogeneity in the full dataset (p = 0.362), and a sensitivity analysis excluding species represented by fewer than three accessions produced a comparable PERMANOVA result (R^2^ = 0.741, p = 0.001), suggesting that the observed species-associated structure was unlikely to be driven primarily by sparse species representation or dispersion heterogeneity. However, partial overlap among accessions sharing identical detection vectors indicated that binary profiles alone were insufficient to resolve all accession-level differences. This pattern suggests that presence/absence profiles are useful for describing species-associated qualitative patterns, whereas quantitative abundance data provide additional resolution among accessions and species. Together, these results support the targeted flavonoid panel as a useful chemical basis for characterizing *Abelmoschus* germplasm.

### 3.4 Principal component structure of targeted flavonoid variation

**Figure 4:**
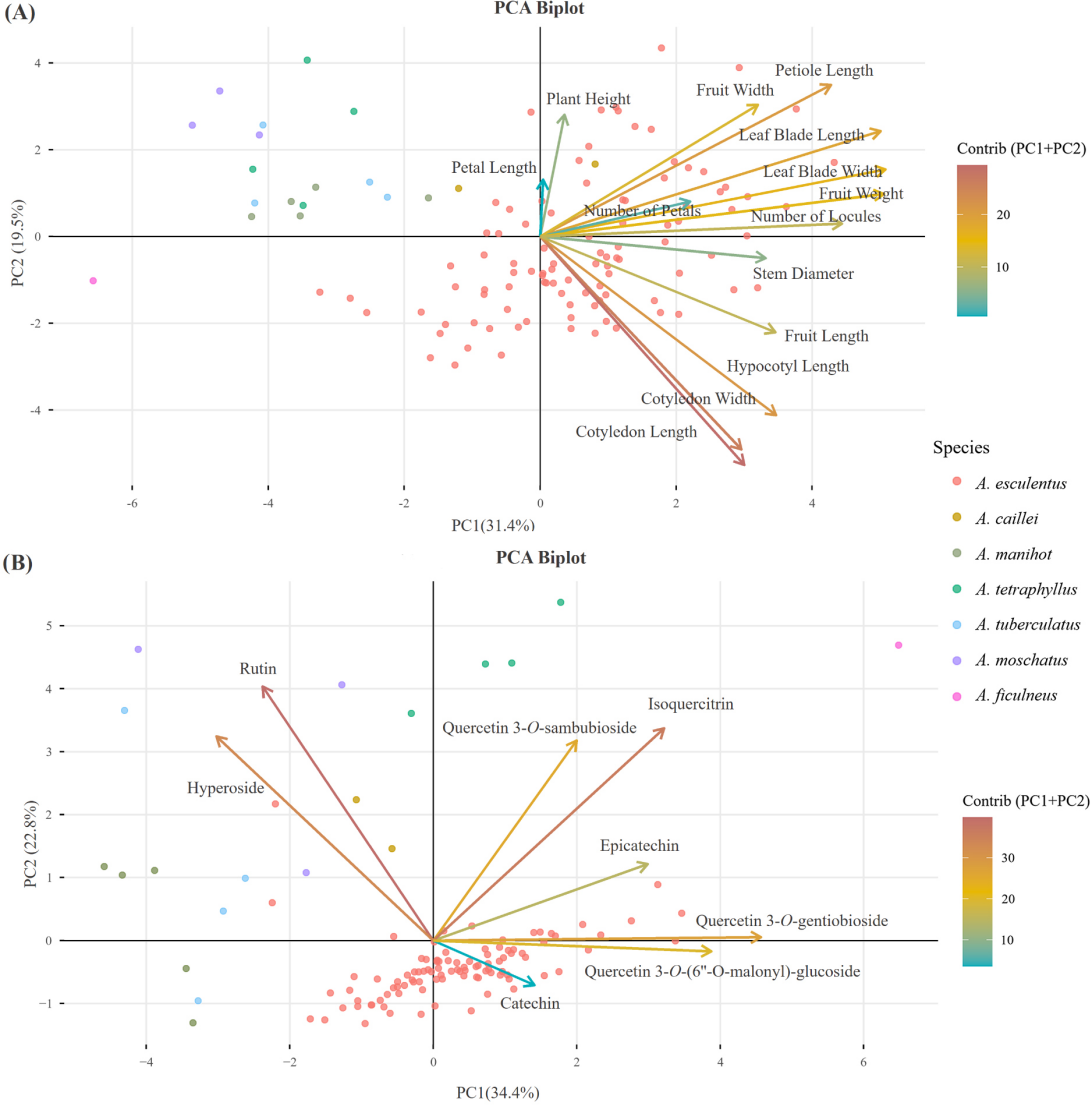
Principal component analysis (PCA) biplots of *Abelmoschus* accessions based on **(A)** 14 z-score-standardized agronomic traits and **(B)** eight z-score-standardized targeted flavonoid concentrations. The points represent individual accessions, which are colored by species. Arrows indicate variable loadings, with directions reflecting the correlations of each variable with PC1 and PC2 and lengths indicating the magnitudes of the loading vectors. Arrow colors indicate the combined contributions of PC1 and PC2 (Contrib. PC1+PC2). In **(A)**, PCA was performed using 14 z-score-standardized agronomic traits: cotyledon length/width, hypocotyl length, plant height, stem diameter, leaf blade length/width, petiole length, number of petals, petal length, fruit length/width/weight, and number of locules (PC1 = 31.4%, PC2 = 19.5%). In **(B)**, PCA was performed using eight z-score-standardized targeted flavonoid concentrations (PC1 = 34.4%, PC2 = 22.8%); detailed loadings and variable contributions are provided in **Supplementary Table 7**.

To explore whether the targeted flavonoid profiles harbor species-associated variation, principal component analysis (PCA) was performed on the z-score-standardized data. PC1 and PC2 explained 34.4% and 22.8% of the total variance, respectively, for a cumulative 57.2% (**Figure 4B**; **Supplementary Table 7**).

PC1, the axis summarizing the principal contrast in targeted flavonoid composition, accounted for the largest share of the overall compositional variance. Its positive direction was defined chiefly by quercetin 3*-O-*gentiobioside (loading 0.524; contribution 27.5%), the malonylated derivative quercetin 3*-O-*(6″*-O-*malonyl)-glucoside (0.445; 19.8%), isoquercitrin (0.369; 13.6%), and the flavan-3-ol epicatechin (0.343; 11.8%), whereas its negative direction was defined by hyperoside (−0.347; 12.0%) and rutin (−0.273; 7.4%). Accordingly, PC1 can be interpreted as the principal compositional contrast axis contrasting profiles in which quercetin 3*-O-*gentiobioside and the malonyl-glucoside are relatively predominant from those with a larger contribution of hyperoside and rutin.

PC2 was strongly associated with rutin, isoquercitrin, hyperoside, and quercetin 3*-O-*sambubioside, all of which loaded in the positive direction (rutin 0.569/32.4%; isoquercitrin 0.475/22.6%; hyperoside 0.458/20.9%; sambubioside 0.448/20.1%). Their combined contribution amounted to approximately 96% of PC2, indicating that its positive direction primarily reflects a compositional pattern in which these flavonol glycosides are relatively higher.

These loading patterns broadly corresponded to the distribution of accession scores by species (**Figure 4B**). *A. esculentus* clustered densely in the region of positive PC1 and low PC2 scores, consistent with a pattern in which gentiobioside and malonyl-glucoside were relatively prominent, whereas rutin and hyperoside contributed less. The two *A. caillei* accessions occupied the negative-PC1, positive-PC2 region, separating from the central *A. esculentus* cluster and lying comparatively close to the other relatives.

The wild relatives were, on the whole, more widely dispersed, suggesting broader compositional variation among the sampled wild accessions. *A. manihot*, *A. tuberculatus*, and *A. moschatus* were distributed in the negative-PC1 region, consistent with the direction in which hyperoside and rutin predominate. *A. tetraphyllus* was located in the region of high PC2 and showed a comparatively distinct distribution, consistent with its measurably higher rutin, isoquercitrin, hyperoside, and sambubioside levels (**Table 1**). *A. tuberculatus* and *A. moschatus* showed a broad spread, suggesting appreciable intraspecific variation despite the limited sample size. By contrast, *A. ficulneus* was positioned alone at the extreme positive end of PC1.

Taken together, flavonoid PCA arranged the accessions along compound-level compositional contrasts. Although some interspecific overlap persisted, several species and accessions showed a partial tendency toward separation—primarily along PC1, which contrasted gentiobioside/malonyl-glucoside-predominant with hyperoside/rutin-predominant profiles, and secondarily along PC2, reflecting higher levels of rutin, isoquercitrin, hyperoside, and sambubioside.

### 3.5 Accession-level compositional grouping patterns based on k-means clustering

**Figure 5:**
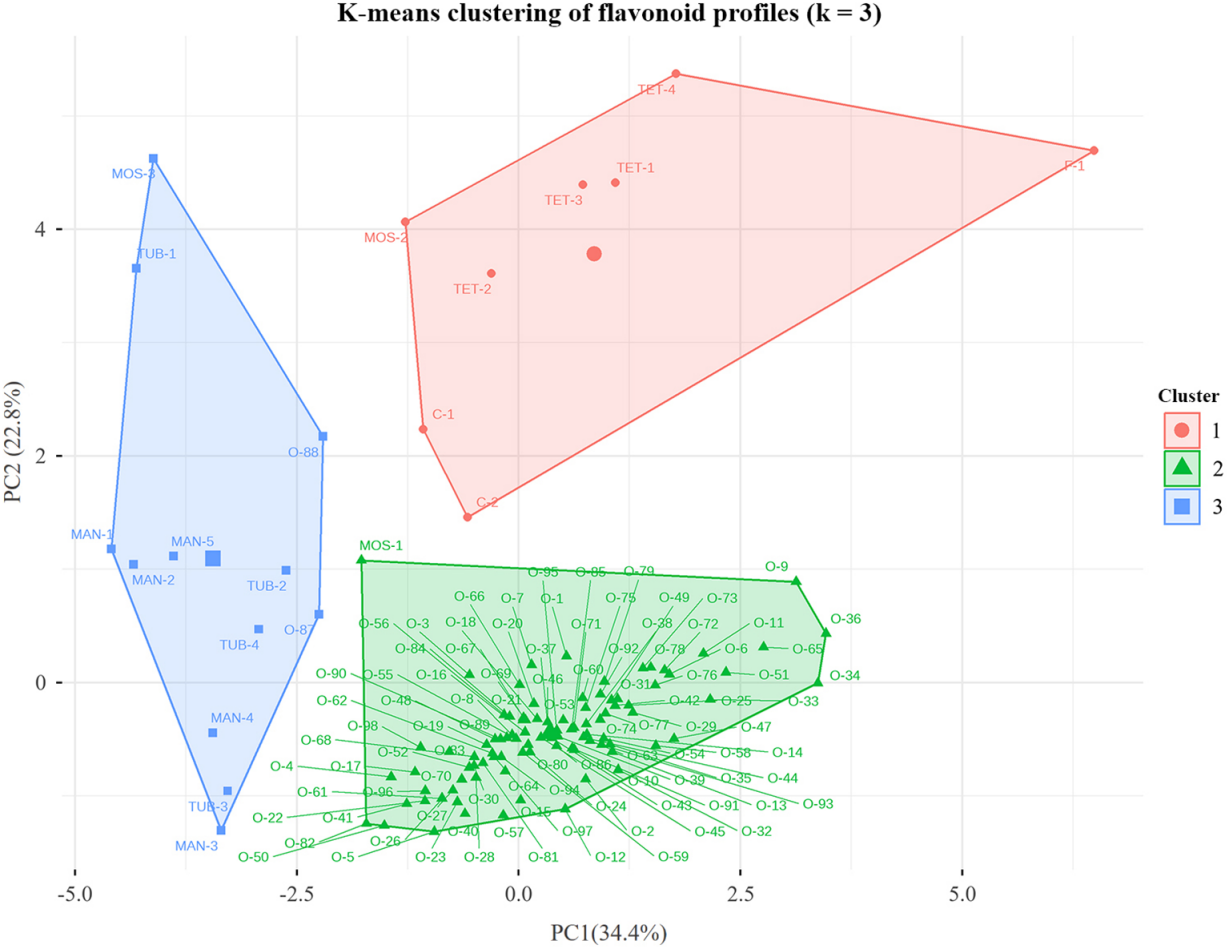
K-means clustering of *Abelmoschus* accessions based on z-score-standardized targeted flavonoid profiles. K-means clustering with k = 3 was performed using the eight standardized flavonoid variables, and the resulting cluster assignments were projected onto the flavonoid PCA score space for visualization. Points represent individual accessions, with colors and symbols indicating k-means cluster assignments. Accession IDs are shown next to the points. PC1 and PC2 correspond to the flavonoid PCA axes shown in **Figure 4B**. The two *A. caillei* accessions were assigned to Cluster 1, whereas the *A. esculentus* accessions O-87 and O-88 were assigned to Cluster 3.

K-means clustering summarized the *Abelmoschus* accessions into three compositional clusters based on z-score-standardized profiles of the eight targeted flavonoids (**Figure 5**). The number of clusters (k = 3) was selected based on the mean silhouette score across candidate values of k. The three clusters were arranged along the main compositional gradients defined by PC1 and PC2, and k-means clustering further summarized this continuous PCA structure into operational accession-level compositional groups (**Figures 4B**, **5**). However, accessions of the same species were not exclusively assigned to a single cluster and were instead assigned according to their similarity in flavonoid composition.

Cluster 2, the largest group, contained most *A. esculentus* accessions and occupied the region around the PCA origin. In particular, this cluster was characterized by a generally low relative accumulation of rutin and hyperoside. In contrast, the two *A. caillei* accessions were both assigned to Cluster 1, separate from the central *A. esculentus* group, despite their agronomic similarity to *A. esculentus*. Cluster 1 occupied the high-PC2 region of the PCA plane and included the two *A. caillei* accessions, all *A. tetraphyllus* accessions, and one accession each of *A. ficulneus* and *A. moschatus*. Cluster 3, located mainly on the negative side of PC1, included all *A. manihot* and *A. tuberculatus* and a subset of *A. esculentus* and *A. moschatus* accessions.

**Figure 6:**
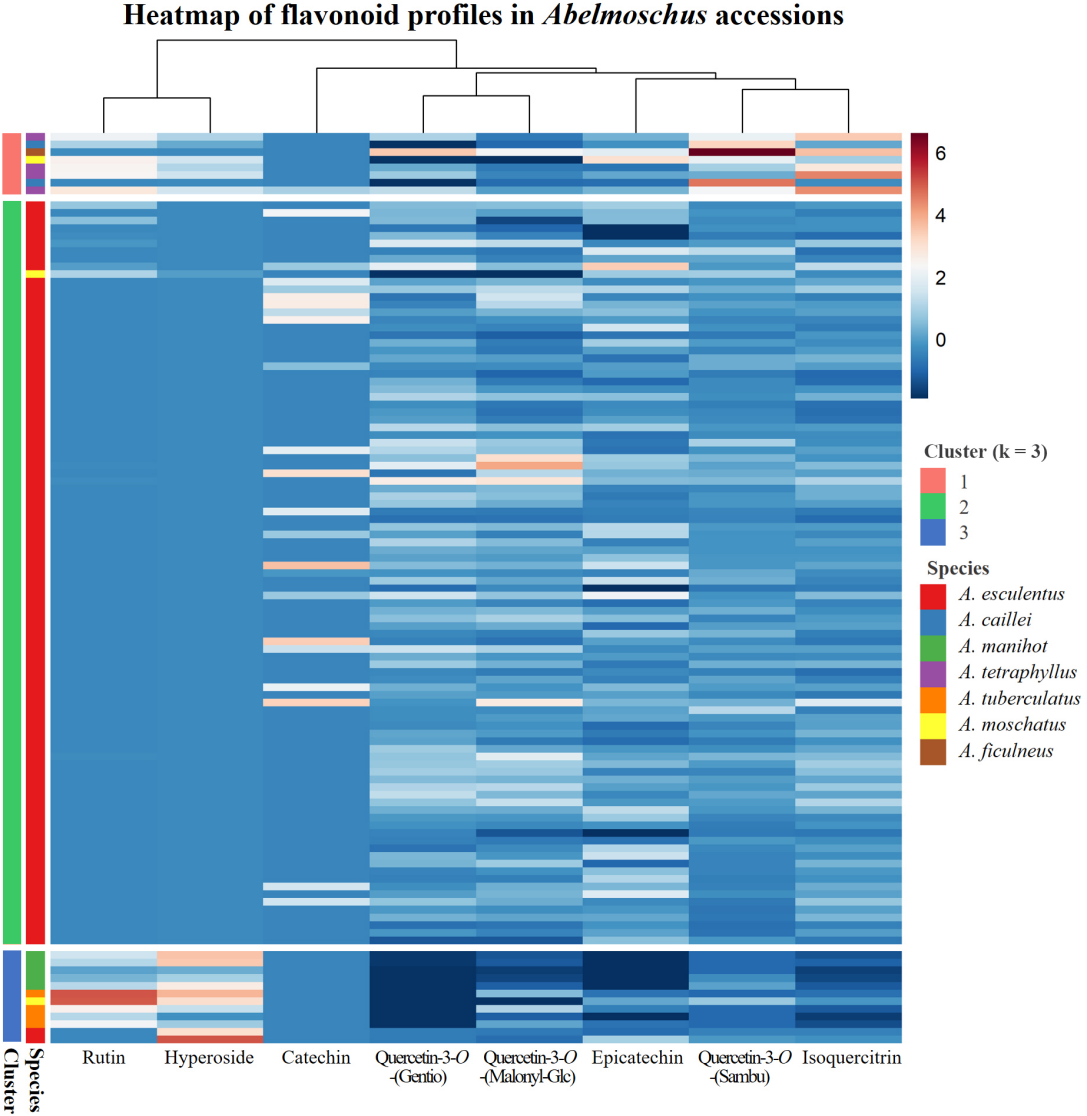
Heatmap of *Abelmoschus* accessions based on flavonoid composition. Values were z-score standardized for each compound. Rows represent individual accessions ordered by k-means clustering (k = 3), with hierarchical clustering applied to each cluster. The columns represent the eight targeted flavonoids, which were hierarchically clustered using Ward’s method and Euclidean distance. Abbreviations: quercetin 3*-O-*(Sambu), quercetin 3*-O-*sambubioside; quercetin 3*-O-*(Gentio), quercetin 3*-O-*gentiobioside; quercetin 3*-O-*(Malonyl-Glc), quercetin 3*-O-*(6′′*-O-*malonyl)-glucoside.

To examine accession-level compositional patterns, a block-ordered heatmap was generated from z-score-standardized values, with the color scale indicating relative accumulation across the accession set (**Figure 6**). The three compositional blocks in the heatmap corresponded directly to the three k-means clusters. This block structure allowed the relative high- and low-accumulation patterns of individual flavonoids to be compared among clusters.

The upper block, corresponding to Cluster 1, showed a relatively high accumulation of quercetin 3*-O-*sambubioside and isoquercitrin. Within this block, isoquercitrin was elevated in *A. tetraphyllus*, whereas quercetin 3*-O-*sambubioside was high in *A. caillei* accessions. In addition, the middle block, corresponding to Cluster 2, consisted mainly of *A. esculentus* accessions and was characterized by low relative accumulation of rutin and hyperoside and relatively high levels of quercetin 3*-O-*gentiobioside. The lower block, corresponding to Cluster 3, showed the highest relative accumulation of rutin and hyperoside, whereas quercetin 3*-O-*gentiobioside, quercetin 3*-O-*(6″*-O-*malonyl)-glucoside, isoquercitrin, and epicatechin were relatively low.

The heatmap helped identify the compositional basis of the atypical cluster assignments observed in several accessions. Within *A. esculentus*, accessions O-87 and O-88 deviated from their species-predominant Cluster 2 and were assigned to Cluster 3, a shift that appears to be reflected by their high accumulation of hyperoside and low levels of quercetin 3*-O-*gentiobioside and quercetin 3*-O-*(6″*-O-*malonyl)-glucoside, matching the rutin/hyperoside-enriched block (**Figure 6**). Similarly, the three *A. moschatus* accessions shared low or undetectable levels of these two quercetin glycosides, yet their distinct quantitative profiles were associated with their assignment to different clusters. MOS-1 exhibited an intermediate pattern. MOS-2 was characterized by elevated levels of epicatechin, quercetin 3*-O-*sambubioside, and isoquercitrin, whereas MOS-3 showed elevated levels of rutin and hyperoside. Meanwhile, the single *A. ficulneus* accession, F-1, showed high relative accumulation of multiple flavonoids, including quercetin 3*-O-*gentiobioside, quercetin 3*-O-*(6″*-O-*malonyl)-glucoside, isoquercitrin, epicatechin, and quercetin 3*-O-*sambubioside, which corresponded with its position on the positive side of both PC1 and PC2 and its assignment to Cluster 1.

In summary, three primary compositional blocks were observed among the *Abelmoschus* accessions: an *A. esculentus*-predominant block with low relative accumulation of rutin and hyperoside, a quercetin 3*-O-*sambubioside/isoquercitrin-enriched block, and a rutin/hyperoside-enriched block. The column dendrogram grouped the same compound pairs that defined the main PCA and heatmap contrasts, including rutin/hyperoside, quercetin 3*-O-*gentiobioside/quercetin 3*-O-*(6″*-O-*malonyl)-glucoside, and quercetin 3*-O-*sambubioside/isoquercitrin.

## 4 Discussion

### 4.1 Chemical profiling extends morphological classification

In this study, agronomic and morphological traits were systematically evaluated, from seedling traits through to vegetative and reproductive organ traits, to characterize interspecific variation within the genus *Abelmoschus*. Quantitative morphological differences among species were observed, particularly in fruit and petal size (**Figure 3**; **Supplementary Table 5**). The fruit and flower images of *Abelmoschus* species presented in this study also provide visual information that complements the species-level characterization (**Figures 1**, **2**).

Nevertheless, even when these quantitative and qualitative variations are considered together, clearly distinguishing all *Abelmoschus* species based solely on external morphology remains challenging. This difficulty partly explains why earlier taxonomic studies had to rely primarily on detailed structural characters, such as the morphology of the epicalyx, calyx, and fine pod surface features, for species delimitation within *Abelmoschus* (Hamon and Van Sloten, 1995; Werner et al., 2016). Morphology-based species identification in *Abelmoschus* requires intensive and expert-dependent phenotyping of fine characters, including the epicalyx and trichomes (Regi et al., 2025).

This limitation was also reflected in the morphology-based PCA in the present study. The cultivated species, *A. esculentus* and *A. caillei*, were broadly separated from their wild relatives mainly along PC1, which was associated with fruit and vegetative organ size (**Figure 4A**). This pattern is consistent with the expected effects of domestication and artificial selection on organ enlargement (Purugganan, 2019). However, the morphology-based PCA showed limited resolution for distinguishing closely related species and for resolving finer differentiation among several wild relatives.

By contrast, PCA based on targeted flavonoid profiles provided complementary resolution in metabolic space (**Figure 4B**). In particular, although the *A. caillei* accessions analyzed in this study shared cultivated-type fruit morphology with *A. esculentus*, they were separated from the main *A. esculentus* distribution in the flavonoid PCA space. *A. tetraphyllus* was also positioned at high PC2 values. *A. manihot* formed a relatively compact cluster toward negative PC1 values and near the PC2 axis, whereas the more variable *A. tuberculatus* and *A. moschatus* accessions were broadly scattered across the metabolic space, resulting in considerable spatial overlap among the wild species and extending into the region occupied by *A. manihot*. In addition, some accessions belonging to the same species were dispersed across different k-means clusters (**Figure 5**).

Morphology-based and flavonoid-based analyses provided partly concordant yet complementary information for interpreting interspecific variation in *Abelmoschus.* This interpretation was consistently supported by Procrustes analysis of the PCA ordinations (r = 0.387, p = 0.001) and by a Pearson Mantel test on Euclidean distance matrices (r = 0.424, p = 0.001), both indicating moderate but significant congruence between the morphological/agronomic and flavonoid datasets. While multivariate analyses of quantitative abundance profiles may have limited resolution for discrete interspecific boundaries, qualitative chemotype profiling may provide additional interpretive resolution. Specifically, the presence or absence of specific flavonoid glycosides may provide an additional basis for evaluating chemical differentiation among the examined *Abelmoschus* species.

### 4.2 Chemotaxonomic significance of flavonoid composition

Beyond the quantitative variation observed in multivariate space, the presence–absence patterns of specific flavonoid glycosides detected by UPLC–MS/MS provided qualitative chemotype signals with potential descriptive value among the examined *Abelmoschus* species.

In particular, the combined detection pattern of two flavonol glycosides, quercetin 3*-O-*gentiobioside and quercetin 3*-O-*sambubioside, provided a useful criterion for classifying the examined species into three chemotype groups. Group I, in which both compounds were detected, included the cultivated species *A. esculentus* and the wild species *A. ficulneus*, *A. manihot*, and *A. tetraphyllus*. Group II, in which only quercetin 3*-O-*sambubioside was detected, included *A. caillei* and *A. moschatus*. In this group, the absence of quercetin 3*-O-*gentiobioside may serve as a candidate qualitative marker distinguishing *A. caillei* from *A. esculentus*, with which it shares cultivated-type fruit morphology, although it does not separate *A. caillei* from *A. moschatus*. In Group III, neither compound was detected in the sampled *A. tuberculatus* accessions. These group-level chemotypes were further refined by species-associated complementary markers. Catechin was detected only sporadically in *A. esculentus* and *A. tetraphyllus*, providing a presence–absence feature associated with these taxa. Epicatechin was not detected in the sampled *A. manihot* accessions, providing an additional distinguishing feature for this species. In addition, quercetin 3*-O-*(6′′*-O-*malonyl)-glucoside was either not detected or present only at trace levels in *A. moschatus* (mean = 0.04 ppm), suggesting the additional chemical distinctiveness of this species.

The qualitative markers described above did not individually resolve all species. For example, within Group I, *A. esculentus* and *A. tetraphyllus* remained similar with respect to the qualitative markers considered in this section. Nevertheless, the quantitative results further supported this qualitative framework. Among the quantified variables, hyperoside and rutin showed the largest interspecific effect sizes, and pairwise differences were mainly observed in comparisons involving *A. esculentus* (**Table 2**; **Supplementary Table 6**). However, because most species other than *A. esculentus* were represented by small numbers of accessions, these patterns should be interpreted as preliminary signals requiring validation with broader geographic and genetic sampling.

The use of specialized metabolite traits, such as sugar moiety structure, acylation pattern, and compound presence or absence, as taxonomic indicators has been reported in several plant groups. In Hibiscus, a genus in the same family (Malvaceae), GC–MS-based phytochemical profiling has been used together with morphological and anatomical traits to support species delimitation (Abdelfattah et al., 2024). As a more direct example involving flavonol glycosides, petal flavonol glycoside profiles and sugar moiety patterns in Rosa have been proposed as chemotaxonomic markers for species- and section-level classification (Sarangowa et al., 2014). In addition, UPLC–QTOF/MS-based metabolomic profiling has been applied to the discrimination of three yellow Camellia species (Zhao et al., 2022), and prenylflavonoid profiles combined with chemometric tools have been used for species differentiation in Epimedium (Bae et al., 2020). The preliminary chemotype signals observed in the present study are consistent with these previous examples, and their taxonomic utility should be evaluated more rigorously through future validation using expanded sampling.

### 4.3 Chemotaxonomic insights for germplasm evaluation and breeding in *Abelmoschus*

The flavonoid chemotypes observed in this study can be interpreted as complementary metabolic information for characterizing closely related *Abelmoschus* taxa and their genetic resources. From a germplasm perspective, the two cultivated species, *A. esculentus* and *A. caillei*, showed clearly contrasting quercetin glycoside profiles: *A. esculentus* was characterized by a quercetin 3*-O-*gentiobioside-dominant pattern, whereas the examined *A. caillei* accessions lacked this compound and instead accumulated quercetin 3*-O-*sambubioside as the predominant glycoside (**Table 1**). Although based on a limited number of *A. caillei* accessions, this contrast indicates that *A. caillei* represents a chemically distinct cultivated gene pool that may serve as a candidate resource for diversifying flavonoid composition in breeding, while also complementing morphology-based identification of these morphologically similar cultivated species (Afzan et al., 2019).

The proximity of some wild species in the flavonoid PCA space further suggests that metabolite profiles may be partially consistent with genetic or phylogenetic structure. For example, the relatively close placement of *A. tetraphyllus* and the single *A. ficulneus* accession was consistent with previous taxonomic evidence suggesting a close relationship between these species (**Figure 4B**; Werner et al., 2016). Similar concordance between metabolite-based clustering and genetic or phylogenetic structure has been reported in tea and cassava germplasm (Yu et al., 2020; Perez-Fons et al., 2023). However, metabolite-based proximity should not be interpreted as direct evidence of phylogenetic relationship. Metabolite profiles can be tissue-specific and may not always correspond directly to genotype-based clustering (Rochfort, 2005; Perez-Fons et al., 2023).

From a breeding perspective, the metabolic variation observed in this study may provide preliminary information for flavonoid-oriented characterization of *Abelmoschus* germplasm. Most *A. esculentus* accessions showed relatively low rutin and hyperoside levels, whereas a small number of outlier accessions were assigned to the high-rutin/high-hyperoside cluster, suggesting that a certain degree of chemical variation exists within the cultivated gene pool (**Figures 5**, **6**). Species-associated differences were also observed among wild relatives, including relatively high isoquercitrin levels in *A. tetraphyllus* and a descriptively high predominance of epicatechin in *A. moschatus* (**Table 1**; **Figure 6**). These results suggest that chemically variable accessions with potential value for improving flavonoid content may exist within *Abelmoschus* germplasm (Bilbrey et al., 2021).

Flavonoid chemotype information may also have practical relevance for germplasm management and taxonomic quality control. In this study, O-10, TET-2, TET-3, and TUB-1 were re-examined because of inconsistencies between their registered species names and observed morphological characters, and they were included in the analyses according to revised taxonomic assignments based on morphological re-evaluation. The targeted flavonoid compositions of these accessions were broadly consistent with the revised morphological assignments (**Supplementary Table 1**), suggesting that metabolite fingerprints can complement morphology-based identification. Similar approaches integrating metabolite fingerprints with molecular or germplasm characterization have been used to support the documentation and quality control of *ex situ* conservation collections (Afzan et al., 2019; Steadman et al., 2022). Therefore, complementary use of metabolite information in *Abelmoschus* may help improve the reliability of species identification and contribute to evaluating the utility of genebank accessions.

Taken together, the targeted flavonoid profiling of *Abelmoschus* germplasm conducted in this study provides useful information for complementing morphological classification and understanding metabolic differentiation among species. In addition, the differences in flavonoid composition observed in several cultivated outlier accessions and wild relatives may provide preliminary criteria for selecting breeding materials aimed at improving functional metabolites. Furthermore, complementary use of metabolite information for germplasm management and taxonomic quality control may help improve the reliability of *ex situ* conservation data and provide baseline information supporting the efficient utilization of *Abelmoschus* genetic resources.

### 4.4 Limitations and future perspectives

Despite these promising findings, this study has some limitations. For instance, the marked imbalance in accession numbers among species, ranging from a single accession in *A. ficulneus* to 98 accessions in *A. esculentus*, may affect the results. This imbalance may have limited our ability to capture accession-level variation associated with diverse geographic and ecological backgrounds, including possible differences in environmental adaptation and flavonoid accumulation. Wild species are known show low germination rates (Hamon and Van Sloten, 1989; Gogoi et al., 2017; Sandeep et al., 2022), and vary in their adaptation to reproductive environments (Marcelline et al., 2024). Cultivated *A. caillei* is also known to show variation in flowering time that is sensitive to day length (Adeniji and Taiwo, 2007; Chinatu et al., 2014). These constraints may partly explain why only a limited number of accessions of some species could be sampled under the temperate growing conditions of this study. Therefore, part of the morphological variation observed under common cultivation may reflect origin-associated adaptation rather than species-level differences alone. Accordingly, for underrepresented species, particularly *A. ficulneus*, the observed morphological and flavonoid profiles should be regarded as accession-level preliminary patterns rather than definitive species-level characteristics.

In contrast, the ambiguous placement of *A. moschatus* in the PCA and k-means analyses is unlikely to reflect sample size alone. This species has been reported to be one of the more polymorphic taxa in *Abelmoschus*, with a contested infraspecific taxonomy, and molecular evidence also indicates heterogeneity among accessions identified as *A. moschatus* (Patil et al., 2015b; Werner et al., 2016). Thus, the unclear compositional grouping of *A. moschatus* may reflect its internal taxonomic and genetic heterogeneity. Resolving this pattern will require sampling designed to capture its infraspecific and geographic variation, rather than simply increasing the number of accessions.

Given that the samples were collected within a single growing season and at a single developmental stage, the possible influence of environmental conditions and post-harvest handling on metabolite variation cannot be excluded. Furthermore, a methodological constraint lies in the targeted nature of our flavonoid analysis. Because this study quantified a predefined set of specific flavonoids, potentially diagnostic compounds outside this panel, such as other specialized metabolite classes or unidentified chromatographic features, were not assessed. Consequently, the profiles identified herein should be interpreted as targeted flavonoid-based profiles rather than comprehensive, whole-metabolome signatures.

To address these limitations, future studies integrating untargeted metabolomics, multi-year and multi-location sampling, and expanded accession coverage for underrepresented species would help test whether the targeted flavonoid-based profiles observed here are stable across environments and representative of broader metabolomic variation.

## 5 Conclusion

Our study revealed that flavonoid profiling can serve as a complementary chemotaxonomic criterion alongside morphology-based classification within the genus *Abelmoschus*. In particular, the predominant patterns of quercetin 3*-O-*gentiobioside and quercetin 3*-O-*sambubioside were associated with species-level differences in the accessions examined and may have practical taxonomic value, although validation is still needed for species represented by limited sample numbers. In addition, the flavonoid profiles generated for wild relatives provide preliminary reference data for future taxonomic studies and genetic resource assessment. Moreover, this study suggests that flavonoid composition can provide complementary information for assessing the identity of morphologically ambiguous accessions. In conclusion, this study provides a targeted flavonoid-based chemotaxonomic framework that may support the evaluation of *Abelmoschus* genetic resources and may inform future breeding strategies and the development of functional materials.

## Data Availability

The raw data supporting the conclusions of this article will be made available by the authors, without undue reservation.

## Supplementary material

Supplementary Figure 1 Principal coordinate analysis (PCoA) of *Abelmoschus* accessions based on binary presence/absence profiles of the eight targeted flavonoids using Jaccard dissimilarity. Points represent individual accessions colored by species.
